# Navigating the crowd: visualizing coordination between genome dynamics, structure, and transcription

**DOI:** 10.1186/s13059-020-02185-y

**Published:** 2020-11-17

**Authors:** Haitham A. Shaban, Roman Barth, Kerstin Bystricky

**Affiliations:** 1 Spectroscopy Department, Physics Division, National Research Centre, Dokki, Cairo 12622 Egypt; 2 Current Address: Institute of Bioengineering, Ecole Polytechnique Fédérale de Lausanne (EPFL), Lausanne, Switzerland; 3 Department of Bionanoscience, Delft University of Technology, 2628 CJ Delft, The Netherlands; 4 Laboratoire de Biologie Moléculaire Eucaryote (LBME), Centre de Biologie Intégrative (CBI), CNRS, UPS, University of Toulouse, 31062 Toulouse, France; 5 Institut Universitaire de France (IUF), Paris, France

## Abstract

The eukaryotic genome is hierarchically structured yet highly dynamic. Regulating transcription in this environment demands a high level of coordination to permit many proteins to interact with chromatin fiber at appropriate sites in a timely manner. We describe how recent advances in quantitative imaging techniques overcome caveats of sequencing-based methods (Hi-C and related) by enabling direct visualization of transcription factors and chromatin at high resolution, from single genes to the whole nucleus. We discuss the contribution of fluorescence imaging to deciphering the principles underlying this coordination within the crowded nuclear space in living cells and discuss challenges ahead.

## Introduction

A largely unexplored question in chromatin biology is how chromatin organization and dynamics relate to function, a question that needs to be addressed in single living cells and at multiple spatio-temporal scales to be answered. DNA transcription, in particular, is a vital but sensitive process to which many players contribute in order to define and maintain cellular identity [1]. These players act through promoter and enhancer sequence elements of one or even several genes. Finding the right partner in a highly crowded environment is a tedious task [2, 3], because multiple enhancers are frequently distant from the promoter of the gene to be regulated along the one-dimensional genome. Furthermore, the motion of chromatin, intrinsically constrained by the mere length of the chain, is hampered in crowded environments [4], making exploration of space a slow process [5]. Recent advances in molecular biology, imaging, and physical and mathematical modeling have improved our understanding of how transcription depends on the nuclear organization and how this organization may facilitate transcription.

Chromatin conformation capture methods [6, 7] are now routinely used to interrogate the chromatin structure of cells in many organisms and in response to various stimuli. However, all sequencing-based methods are by nature invasive and thus make microscopy-based single-cell time course experiments a necessity to understand the dynamic behavior of chromatin. For a comprehensive comparison between different imaging techniques used to studying genome organization and transcription, we refer the reader to recent reviews [8–11]. In addition, imaging techniques can be multiplexed for simultaneous visualization of a multitude of chromatin constituents and are amenable to high-throughput analysis of genome organization at kilobase resolution [10]. While super resolution imaging-based approaches are often performed in fixed cells, time-resolved whole-chromatin imaging recently revealed that chromatin moves in a spatially and temporally correlated manner at a nanoscale resolution [12, 13]. Such correlated motion might be caused by active mechanisms [14], including transcription [15, 16] and/or polymer properties [17]. In silico, physical modeling of data from chromosome conformation capture techniques and fluorescence imaging produced several models which describe genome organization in the context of biological processes such as transcription [18, 19]. Comprehensive time-resolved data complemented with static and ensemble data now bear great promise to correlate changes in the 3D organization to transcription.

Here, we describe how recent advances in live-cell imaging of chromatin in eukaryotic cells enriched our understanding of genome organization at different spatial and temporal scales. We start by considering the complementary views that well-established and yet continuously developing sequencing-based “C”-methods (henceforth Hi-C) and imaging-based methods can offer. We then outline various strategies to interrogate chromatin structure and/or dynamics in three-dimensional space and time, from a single gene to the complete genome, and describe how transcription activity influences chromatin folding and dynamics. We finally discuss how physical principles can explain the spatio-temporal coordination between the chromatin structure and dynamics and transcription in the nucleus.

## Fluorescence imaging and Hi-C: complementary and controversial views

Hi-C revolutionized our knowledge of genome structure: a hierarchy of structural elements was discovered, the most prominent ones being DNA loops, topologically associated domains (TADs), and compartments [20–22]. While those elements are derived from a population average of structures, single-cell Hi-C has confirmed what cell biologists have known for decades that chromosome and chromosome domain conformations vary from cell to cell [23–27]. Distance measurements between FISH-labeled loci can validate selected features seen in models established from Hi-C maps [28–30], but distances do not necessarily correlate with contact frequencies [27, 31, 32], a tendency confirmed by high-throughput approaches [33, 34]. Furthermore, it is tedious to probe distances throughout the whole genome since FISH probes must be designed for each region of interest and can thus detect only a subset of chosen genomic loci in a reasonable time.

**Fig. 1:**
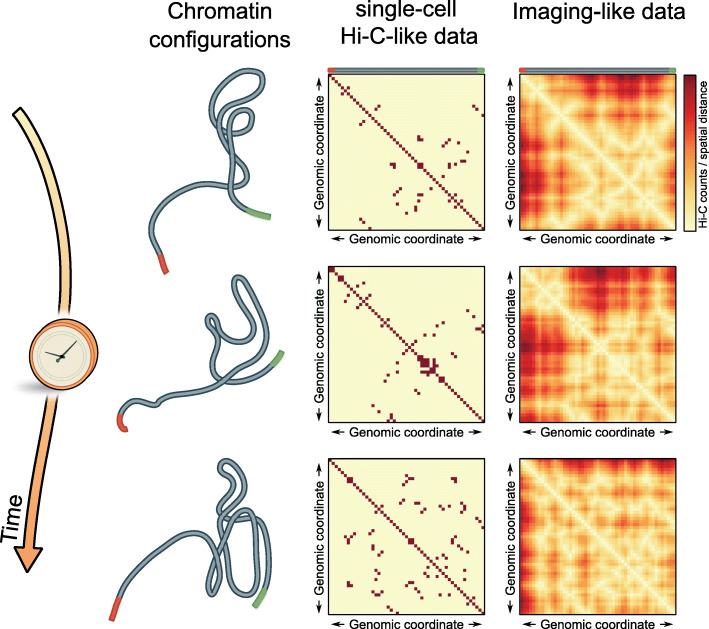
Advances in imaging techniques allow rich information about chromatin beyond Hi-C. Chromatin is constantly remodeled during time (top to bottom), which is illustrated on the basis of a short polymer which changes its configuration over time (left). A single-cell Hi-C-like type of data over time of this polymer would reveal relatively few contacts at each time point for single cells as the technique relies on proximity ligation (middle column). In contrast, imaging offers the determination of actual positions and distances between any two loci in three dimensions and thus reveals a more complete picture than “C” methods (right column). The illustrative maps were created by tracing the contour of the polymer shown on the left and computing the pairwise distance between any two loci which is shown in the imaging-like matrix. The Hi-C map is a thresholded version of the distance map and shows contacts only at small spatial distances. Yet, note that there may be a broad distance distribution underlying measured Hi-C contacts [36], and as such, the illustration is highly simplified. The Hi-C map is a thresholded version of the distance map and shows contacts only at small spatial distances. While imaging chromatin at many loci simultaneously is currently, with a few exceptions, done in fixed cells, it has the potential to advance toward analysis in living cells in the future. However, a single-cell time evolution of chromatin structure by Hi-C cannot be obtained since Hi-C is a destructive method

Hi-C and derived methods can assess the probability of crosslinking which is a measure of the frequency that chromatin loci are in proximity, probing spatial distances in the range of ~ 400–600 nm [35]. By imaging, it is possible to quantify the distribution of actual spatial distances between any two or more loci (Fig. 1), making “C” and imaging complementary approaches [37]. Imaging also allows to characterize the shape of a given genomic region of interest [38] and to monitor the positioning of genes relative to nuclear compartments which can be visualized simultaneously [33, 39, 40] (Fig. 1).

**Fig. 2:**
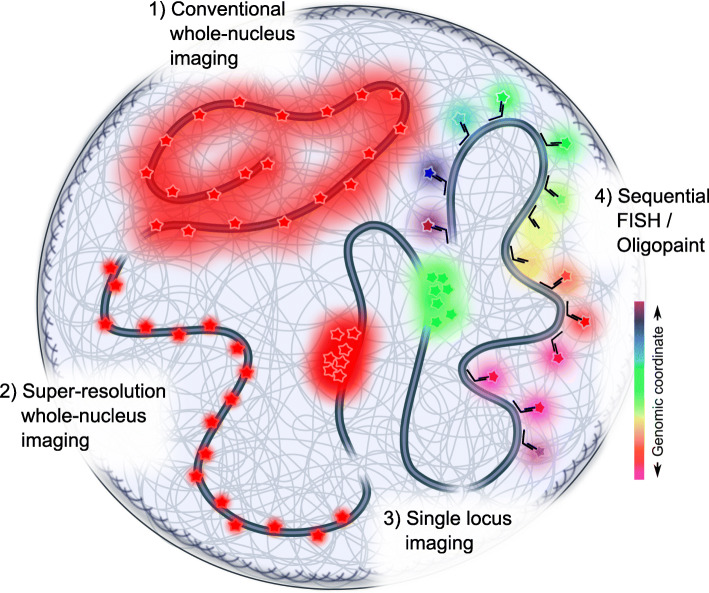
Labeling and imaging strategies to image chromatin. Conventional labeling using stably expressed fluorescent proteins or organic dyes usually covers the whole genome unspecifically (modality 1). Due to the high density of chromatin, only a spatial resolution well above the diffraction limit can be achieved, while fast imaging is generally possible. Super-resolution imaging of chromatin overcomes the diffraction limit but is challenging since chromatin in vivo is not well structured (modality 2). Usually, resolution is gained for the expense of acquisition time and thus cells must be fixed. While conventional and super-resolution whole-genome labeling is sequence-unspecific, single loci can be targeted using fluorescence in situ hybridization (FISH; in case of fixed cell imaging), ANCHOR, a CRISPR/Cas9 system, or others (modality 3). Single locus labeling can be done in fixed as well as in living cells. To cover an extended region of chromatin for which sequence information is available, a sequential FISH or oligopaint strategy can be followed in fixed cells (modality 4). Using oligonucleotides which are designed for the specific genomic region, the chromatin configuration is sampled by sequential hybridization rounds and computationally reconstructed

With the advent of sequential FISH [31], the three-dimensional folding of chromatin regions was probed directly in single cells and in situ (Fig. 2). Cooperative three-way interactions between loci [41] not seen previously using population-averaged contact maps were identified. Imaging further showed that TADs appear as discrete nano-compartments, which are spatially arranged side by side [41–43], although contact frequencies between loci within TADs only slightly (~ 2-fold) increased compared to loci within neighboring TADs [27, 44]. Integrating multiplexed imaging of RNA [45, 46] with sequential DNA FISH [42, 43] revealed that genes located at TAD boundaries were transcribed more frequently than genes within the TAD’s interior highlighting that features of local chromatin structure and transcription activity are related.

Despite their success, three-dimensional chromatin structures derived from either sequential DNA FISH imaging or reconstructed from Hi-C maps are currently limited to (i) fixed cells and (ii) a genomic resolution of ~ 2 kb [43], although sequencing-based techniques to map contacts at the nucleosome level are being developed [47, 48]. As an alternative approach, sequence-unspecific super-resolution imaging of histone H2B and of histone post-translational modifications was employed to visualize chromatin in fixed [49, 50], and recently also in living, cells [12, 51] (Fig. 2). These approaches revealed that nucleosomes transiently associate in groups. Since the genome and associated processes are inherently dynamic and many DNA-proteins as well as DNA-DNA contacts are transient, fixed cell studies naturally cannot capture this process and population assays represent a blurred picture over many cells. Time-resolved imaging is thus needed to appreciate the dynamic nature of the biological processes such as transcription in single cells. However, it is also evident that imaging specific sequences is necessary to place time-course data into a functional context. In the future, labeling strategies will need to be designed for fluorescence live-cell imaging to simultaneously observe structural changes of chromatin and its functional consequences simultaneously in the same cell (Fig. 1).

## Chromatin dynamics from single genes to the entire nucleus

### Motion of individual chromatin loci is subdiffusive in living eukaryotic cells

To gain insights into the dynamic behavior and spatio-temporal organization of chromosomes in live cells, distinct genomic sites can be labeled and tracked in real time (Fig. 2). Several methods have been developed to create a fluorescent signal distinguishable from background fluorescence [52]. Labeled loci can then be tracked over various length and time scales using single-particle tracking (SPT) approaches [53]. Gene editing tools contributed largely to visualize chromatin. The first method to tag chromatin loci and monitor their motion in real time used a chimeric DNA binding lac repressor - GFP fusion protein targeted to multiple repeats of operator binding sites [54, 55]. This approach showed that chromatin motion is constrained and anomalous and influenced by nuclear structures, cell cycle, and function [55–57]. A more innocuous system suited to study changes in chromatin motion in close vicinity of a genomic locus without disrupting its function is the ANCHOR/ParB DNA-labeling system. ANCHOR implants a short non-repetitive sequence near the gene of interest which triggers the accumulation of fluorescent ParB proteins [58, 59]. Several editing-free systems such as transcription activator-like effectors (TALE) [60, 61] and clustered regularly interspaced short palindromic repeats (CRISPR/inactive dCas9) [62, 63] have also been used to visualize genomic loci [62–64]. These systems frequently suffer from poor SNR, but tracking of repetitive DNA sequences which provide a large number of sites confirmed that their motion is also constrained [62, 63, 65]. Most of these SPT studies concluded that the majority of mammalian interphase chromatin motion is subdiffusive [59, 66–68] and in some cases super-diffusive [69, 70].

### Motion of individual chromatin loci near genes changes in response to transcription activation in living eukaryotic cells

The prevalence of inactive genes to localize near the nuclear periphery and active genes to prefer the nuclear interior suggests that those loci undergo long-range movements upon gene activation toward the nuclear interior [69]. This can however occur over hours or days, during development and differentiation, and cannot inform on the underlying physical properties of the chromatin fiber (for review see [71]). To determine how activating transcription influences the local motion of chromatin, a few recent studies have examined the behavior of tags inserted in the vicinity of genes. Combining the ANCHOR DNA-labeling system with MS2-labeled mRNAs enabled monitoring the motion of a single transgene under control of a hormone inducible endogenous promoter before and after transcription in the same cell [59]. While the motion of the single-gene domain remained subdiffusive, transcription initiation caused local confinement of the gene within seconds only when mRNA was produced in the analyzed cells [59]. Confinement was maintained during elongation by RNA polymerase II (RNAP II). These findings suggest that increased chromatin collisions and assembly of functional protein-DNA complexes during the steps of transcription reduce motion, coherent with an increase in local crowding (discussed below). An imaging system based on chimeric arrays of gRNA oligonucleotides (CARGO) to which dCas9-GFP fusion proteins bind was applied to monitor the *Fgf5* enhancer and promotor loci in mouse embryonic stem cells (ESC) before and 4 days after inducing cellular differentiation [64]. In contrast to the study in mammary tumor cells by Germier et al. [59], the study by Gu et al. [64] reported that mobility increases at the enhancer and promoter of the target gene when its activation was inhibited [64]. The increase in calculated diffusion coefficients is explained by non-thermal agitation of chromatin domains, which may favor enhancer-promoter contacts as a result of the stochastic confrontations within the TADs [64]. However, the establishment of a TAD as defined by ensemble approaches does not necessarily insulate from inter-TAD contacts in single cells and is thus unlikely to promote enhanced contact frequencies upon enhanced dynamics. Contacts (within a physical distance of < 10 nm) of two loci can thus only be reliably enhanced at loop bases due to the repeated extrusion of the region governing the loci of interest into loops, not because TAD formation promotes contacts of two loci within TADs at any time. It is also possible that changes in global chromatin organization in ES cells before and after 4 days of inducing differentiation have different dynamic properties independently of the motion of any individual gene or regulatory locus. This has yet to be tested. While recent studies of chromatin dynamic imaging at single nucleosome and global chromatin levels support the finding that chromatin is confined in transcriptionally active nuclei compared to cells treated with RNAP II inhibitors [16, 72], studying single gene motion in different cell lines (e.g., human cancer cells vs mouse ESC) using the same experimental setup and analysis method would be important.

### Chromatin dynamics are spatially partitioned in nuclear domains

Early tools for visualizing chromatin within the entire nucleus either using the expression of fluorescent histones or by incorporating transfected fluorescent nucleotides during replication [73, 74] contributed later for studying the global dynamics of the genome. The alliance between photo-activated localization microscopy (PALM) and SPT is a powerful tool for quantifying chromatin dynamics at the single nucleosome level for sparse domains [16, 75]. Relying on the photoactivation of PAFP fluorophore-tagged histone H2B, this method tracks unbleached nucleosomes for up to 500 ms [75]. However, longer acquisition time is required for accurate analysis and in-depth understanding of the processes governing nucleosome dynamics at the physically and biologically relevant time scales. New organic fluorophore tags of DNA such as Hoechst 33342 and its far-red variants (silicon-rhodamine-Hoechst) [76, 77] have been introduced to circumvent phototoxicity and photobleaching that enabled analyzing the entire chromatin in vivo for up to 30 s [15, 72].

Imaging abundant nuclear (macro-) molecules such as chromatin, transcription factors (TF), or RNA poses limitations on the application of common methods such as SPT to analyze their dynamics. Particle identification in a densely labeled environment is not always unique and may even be impossible due to the limited spatial resolution. As a complement to SPT for studying chromatin motion, raster image correlation spectroscopy, pair correlation analysis, and optical flow-based methods have been applied to quantify global chromatin motion in living cells [15, 67, 78, 79]. The high-resolution diffusion mapping (Hi-D) method reconstructs trajectories at nanoscale precision across the entire nucleus simultaneously [72]. A Bayesian inference approach is used to infer types of diffusion for every single pixel. Consequently, the resulting high-resolution maps of global chromatin diffusion provide biophysical parameters (type of diffusion, diffusion constant, anomalous exponent) of different nuclear domains in single living cells [72]. Chromatin domains spanning 0.3–3 μm positioned in a mosaic-like manner were detected in the nuclear interior. These domains were seen to be remodeled in response to transcriptional activity. Strikingly, the dynamic properties of these patterns were uncoupled from chromatin density [72]. Emerging whole-chromatin super-resolution techniques could detect nanodomains, consisting of up to ten nucleosomes, as a small-scale structural entity (diameter < 100 nm) of the genome [49, 51] and further developments enabled to determine whole-chromatin chromatin dynamics well below the diffraction limit to link structural changes of these entities to dynamics [12]. Mapping nuclear dynamics of dense molecules across the entire nucleus simultaneously instead of the isolated visualization of single loci opens new perspectives for our understanding of genome motion in the context of nuclear architecture.

## Spatio-temporal organization of transcriptional dynamics

Transcription is a highly regulated and dynamic process [80] and is set up by transcription initiation controlled by binding and release of TFs to DNA sequences and transcriptional machineries [81, 82]. TF-binding kinetics and diffusion within the target DNA domains in live cells were originally determined using fluorescence recovery after photobleaching (FRAP) [81, 83]. Later, improving the signal to noise ratio (SNR) of labeled molecules enabled detecting and quantifying binding events of TFs to DNA in two [84] and three dimensions [85]. Fluorescence microscopy methods such as FRAP, fluorescence correlation spectroscopy (FCS) [86], SPT, and super-resolution microscopy have greatly contributed to uncover and quantify the variability of TF dynamics [87], including interactions with their genomic targets by measuring the residence times, on/off rates, and diffusion constants [80, 88]. The majority of these studies showed that the residence time of TFs at specific and non-specific binding sites varied by some orders of magnitude, with longer residence time for specific binding (milliseconds to a few seconds) than for non-specific binding (tens to hundreds of milliseconds) [84, 89]. The residence time of bound TFs may alter the spatio-temporal expression patterns, dynamics, and nuclear organization of TF-loaded loci in live cells [90]. Specifically, swift TF release is required for regulating expression patterns. Nonetheless, long residence times were recorded at some sites, making the process less tunable [80, 91, 92]. Long-time imaging of the TF Sox2 and chromatin domains recorded jumps of Sox2 between binding sites suggesting that kinetic chromatin domain structures facilitate transcription regulation at this locus in embryonic stem cell [87, 93]. Yet, contacts between Sox2 and its enhancer, if any, were too short lived to be imaged [94] in an engineered system. Simultaneous imaging of labeled promoter, enhancer, and/or gene sequences in cells in which the local transcriptional state is known could in the future unequivocally characterize relative positions, possible contacts, and their frequencies between these transcriptional elements and sharpen our understanding of their coordination from transcription initiation to termination.

Transcription is not only regulated in time but depends largely also on the spatial organization of TFs and RNAP II [95]. Furthermore, transcription can modify the architecture and dynamics of DNA [96]. Nuclear gene positioning, packing, and looping of transcriptionally active loci are dynamic, granting proteins access to regulate the transcription processes at different levels [2, 97–99]. Likewise, the spatial distribution of TFs and RNAP II is highly imparted upon transcription initiation; for instance, RNAP II was found to cluster in transcription factories in early fluorescence and electron microscopy studies [100–104]. These transcription factories may form anchor points to which chromatin is tethered, thereby co-regulating the dynamics of chromatin and proteins that compose the factories [16]. Indeed, the motion of global chromatin and RNAP II correlates when examined at a nanoscale dynamic resolution and a time resolution of 5 fps [72], and chromatin dynamics are locally [59] and globally [16, 72] confined upon transcription. Furthermore, the formation of correlated chromatin domains could be linked to transcriptional activity. The correlated motion was decreased but not lost completely by blocking RNAP II activity [15, 67]. These results indicate that stopping transcription at early stages does not eliminate long-range contacts and does not dissolve transcription factories [105, 106]. Spot tracking analysis of the even-skipped (eve) locus promotor and enhancer during transcription in live *Drosophila* embryos identified three topological states: an open state which is however inactively transcribed and a paired *homie–homie* state which can be both actively and inactively transcribed [107]. Accumulation of nascent mRNA indicates that transcription initiation transiently enhances the stability of the proximal configuration between enhancer and promoter and increases the gene’s spatial compaction, consistent with hindered motion [59, 107, 108]. These examples highlight the role of transcription in shaping the hierarchical organization of mammalian genomes in the nuclear space as well as their dynamics.

## Mechanisms governing the dynamics of the genome during transcription

Several mechanisms and models were proposed to explain the nature of the observed chromatin dynamics. Here, we discuss the most popular and experimentally—by fluorescence microscopy and biochemical methods—well-established principles.

### Liquid-liquid phase separation as a physical model to understand the regulation of gene expression

**Fig. 3:**
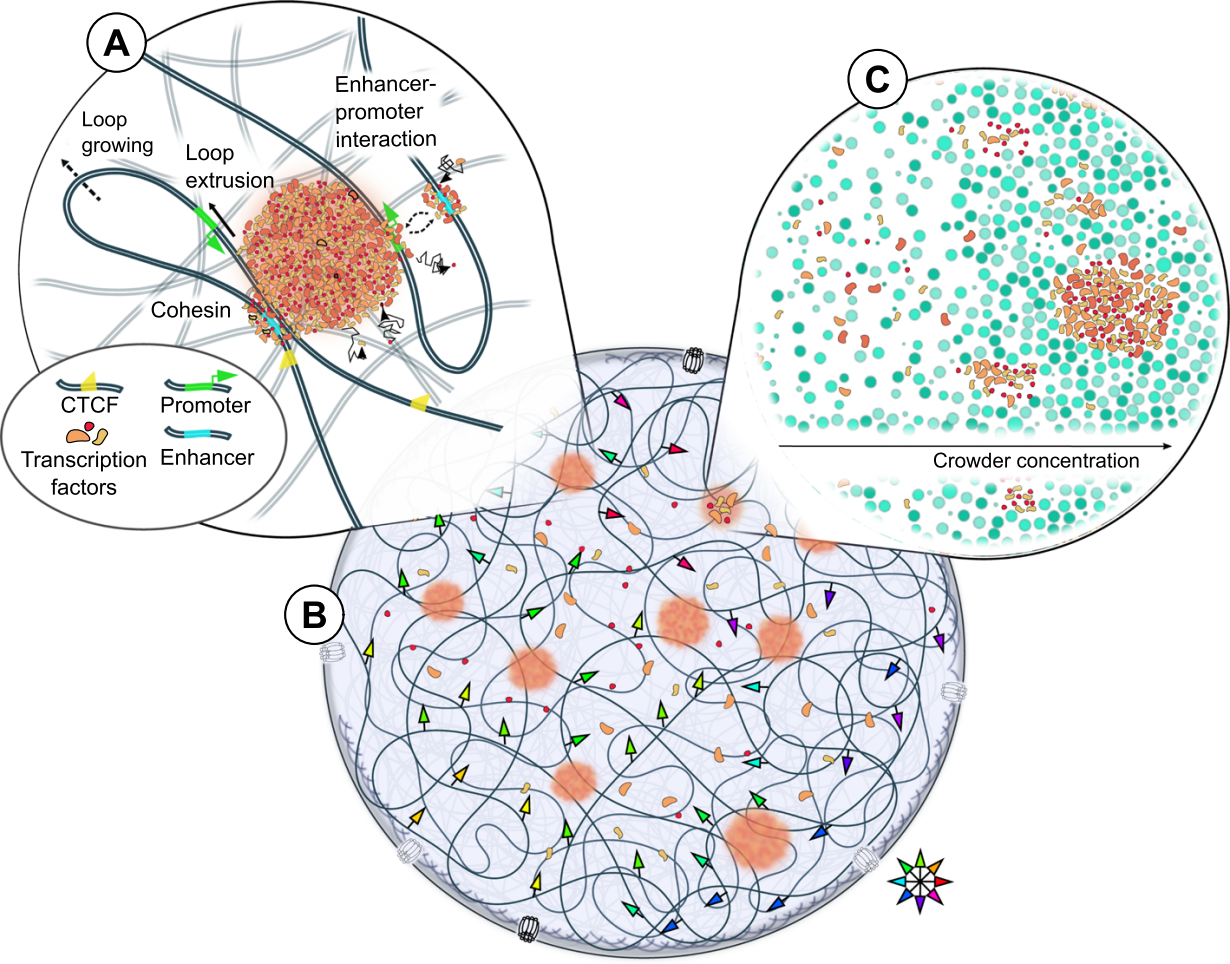
Mechanisms for chromatin organization and dynamics during transcription. (A) The transcriptional hubs are formed by liquid-liquid phase separation of transcription factors, which in turn is mediated by the local crowding conditions. TFs binding to both enhancer and promoter mediate enhancer-promoter contacts by an effective attraction exerted by the LLPS property of transcription factories. Once transcription is initiated by these enhancer-promoter contacts, transcription elongation proceeds by reeling the transcribed gene body along the transcription factory. Loop extrusion by cohesin can additionally establish transient enhancer-promoter contacts, and the turnover time of cohesin and CTCF regulates the frequency of these contacts. Loop extrusion dynamics and the placement of (semi-) permeable border elements may thus regulate transcription. (B) The nucleus is sprinkled with transcription factories to which chromatin is tethered. The resulting network of transcriptional hubs restricts chromatin motion and induces a stiffness to chromatin, which is expressed in long-range correlations of chromatin dynamics (colored arrows). (C) The local molecular crowding reduction of chromatin mobility upon transcription. This transition is associated to high molecular crowding, eventually facilitating the formation of transcription factories to which chromatin is tethered

Upon transcription initiation, hundreds of proteins have to reach a transcription start site in a highly coordinated manner [109]. Live-cell super-resolution imaging revealed that RNAP II engages transiently in transcription factories during transcription initiation [110, 111] (Fig. 3 (A)). The mechanism by which transcription factories form could be resolved by lattice light-sheet imaging in living cells: transcription factories behave like liquid condensates [112], membrane-less compartments, likely formed spontaneously by a liquid-liquid phase separation (LLPS) mechanism due to spatial concentration heterogeneities of the condensate components [113]. These findings found support [112, 114–119] in that the carboxyl-terminal domain (CTD) of RNAP II, an intrinsically disordered low-complexity region, can undergo cooperative LLPS in vitro [117]. The condensates are dissolved upon CTD phosphorylation [117], which is the same mechanism by which RNAP II is braced for transcription elongation [118, 120]. By controlling CTD phosphorylation, the contact duration between transcription factories and DNA may thus be regulated [110, 121]. This mechanism appears to be common to several other transcription factors in vivo [119], which led to the current view that transcription factories could be condensates in vivo, whose intrinsically disordered low-complexity sequence domains undergo liquid-liquid phase separation (LLPS) (Fig. 3 (A, B)).

Super-enhancers, clusters of putative enhancers in close proximity with unordinary high density of transcriptional co-activators binding [112, 115, 116], have shown the potential to form a nucleation point for LLPS, directed by master TFs [122]. The unusually high transcription activity of super-enhancers has to be “contained” within insulated neighborhoods, demarcated by Hi-C-defined TAD boundaries, in order to specifically activate their target gene [122]. As such, the local chromatin architecture appears to be vital to prevent ectopic gene activation.

LLPS does not only concern protein condensates such as transcription factories but may also interact with and actively shape the surrounding chromatin environment [123] (Fig. 3 (B)), in particular, at super-enhancers [124, 125]. For instance, the fusion of condensates minimizes their surface tension and deforms chromatin locally which may bring chromatin loci in the vicinity of each other [123]. Merging of condensates of TFs at promoters and enhancers could explain how these potentially distant genomic regions find each other (Fig. 3 (A)). Tethering of chromatin to transcriptional hubs could also account for reduced freedom of chromatin movement [16], and result in a stiffer DNA network, which is detected by spatially coherent motion [15, 67] (Fig. 3 (B)). Furthermore, chromatin itself can self-organize and phase-separate, consistent with entropic forces of polymers [126]. For instance, heterochromatin is preferentially found at the nuclear periphery and near nucleoli, and repressed genes are found buried inside nuclear compartments, while active genes and RNA-associated proteins preferentially lie on the surface of chromatin domains [51, 127]. Fluorescence microscopy is an excellent tool to detect and quantitatively study such LLPS-driven chromatin bodies and compartments [128].

### Molecular crowding drives chromatin dynamics, confinement, and function

Molecular crowding significantly influences the diffusion of proteins within the nucleus and is a driver for transcriptional silencing within heterochromatin by phase separation [4, 129, 130]. Crowding affects the binding affinity of RNAP II on DNA [131, 132] such that in vitro transcription is markedly enhanced only when crowder volume fractions resemble those found in vivo [133, 134]. Especially temporal changes in crowder density, as opposed to a commonly assumed steady-state crowder concentration, have been shown to be able to selectively up- or downregulate genes and could thus constitute an additional gene regulatory pathway for the cell [135]. Because the microenvironment of a chromatin locus relates to its dynamics [136–138], such crowding density fluctuations may be captured by varying chromatin dynamics over time. The reduction of chromatin mobility upon transcription suggests that this transition is related to a local accumulation of crowders, eventually facilitating phase separation of transcription factors to which chromatin is tethered [16, 72] (Fig. 3 (C)). Finally, it should be noted that not only proteins, but also chromatin itself, is a crowding agent. The observation that LLPS takes place in regions of low chromatin occupancy [123] could relate to the fact the local crowder concentrations can control the occurrence of LLPS. It is likely that several physical forces resulting from macromolecular crowding, chromatin compaction, and LLPS cooperate in order to spatially regulate transcription [139, 140].

### Coherent motion of chromatin depends on enzymatic activity

Chromatin was shown to move coherently over several microns by live-cell imaging. This coherent motion was modulated by activating DNA processes [15, 67] (Fig. 3 (B)). Computational models of this motion suggest that chromatin is dynamically partitioned [5, 17]. Intriguingly, boundaries of coherently moving domains appeared uncoupled from domains of similar chromatin compaction, but not from transcription states [15, 72]. Self-organizing properties of active systems, propelled by ATP-driven proteins and promoted by the long-scale correlation due to hydrodynamic interactions of the activity-induced motion, could be at the origin of coherence [14, 67, 141–143]. However, it should be pointed out that qualitatively similar findings can be yielded computationally when only chromatin chain conformation and epigenetic marks are taken into account [5, 17, 144]. Thus, the role of active chromatin remodelers remains to be further evaluated. Combining quantitative fluorescence imaging, Hi-C, and computational polymer modeling to study the mobility of yeast chromosomes demonstrated how heterogeneous binding patterns of proteins along the chromatin fiber could lead to thermodynamically driven self-organization and differential mobility [145]. Thus, while transcription or other energy-consuming processes can induce coherent motion, additional players shaping chromatin architecture and epigenetic landscapes should not be overlooked when interpreting live-cell imaging data of chromatin.

### Loop extrusion can create windows of opportunity for fine-tuning transcription

The *transcription factory model* has been proposed to explain the mechanisms leading to genome folding [18, 93, 146, 147]. Binding of TFs to DNA induces a positive feedback loop by quickly re-binding dissolved TFs and recruiting new ones from the pool of freely diffusing TFs [18, 146] (Fig. 3 (A)). The resulting TF condensate is able to bridge different DNA regions and causes an effective bridging-induced attraction [146]. Merging of clusters at different DNA segments establishes chromatin loops, whose loop bases colocalize with TFs. This relatively simple mechanism was shown to be sufficient to recapitulate many features of chromatin structure established by Hi-C such as rosettes and TADs [148, 149]. This transcription factory model [18] supports the concept that transcription is an important driver of chromatin folding and dynamics [1, 15, 16, 72, 96], but also takes into consideration chromatin contacts mediated by proteins which are unrelated or only distantly related to transcription.

The *loop extrusion model* [150–152] conceptualizes how chromosome structure emerges due to the extrusion of DNA loops from structural maintenance of chromosomes (SMC) proteins [153–157] as loop extruding factors. Based on a collaboration between the SMC protein cohesin and molecular boundary elements (CCCTC-binding factor (CTCF) proteins), the loop extrusion model recapitulates the hierarchical organization of chromosomes revealed by Hi-C [158, 159]. Nevertheless, the model fails to reflect experimentally observed large-scale interactions [160], and it is now clear that phase separation mechanisms go hand in hand with loop extrusion [161].

In contrast, the transcription factory model poorly predicts details of interactions but correctly captures long-range contacts [160]. A combinatorial model consisting of the transcription factor and loop extrusion model faithfully reproduces many Hi-C features suggesting that TFs and cohesin have complementary roles in genome organization [160]. Chromatin domain boundaries are often found at long and highly expressed genes, whose strength is driven by transcription length and transcription rate [162], and most chromatin contacts are established between active transcription units [163, 164]. Furthermore, increased contact frequencies in *trans*, at the expense of *cis* contacts, as revealed by ChiA-PET [165], can be explained by the transcription factory model because this model is compatible with the idea that active genes on different chromosomes could be co-transcribed in the same transcription factory.

The loop extruding activity of cohesin was recently illustrated by in vitro experiments [157, 166, 167]. Given this mechanism, transient contacts between two genomic loci can be established, in particular enhancers and promoters, during the growth of the loop. The bulky transcriptional machinery is likely to influence the position of cohesin-mediated DNA loops [96, 168] (Fig. 3 (A)). However, how cohesin interacts with single or multiple stalled or elongating transcription complexes remains to be interrogated experimentally.

The loop extrusion and transcription factory models appear to complementarily explain how the genome is dynamically organized. Both models imply that transcription can influence the genome organization and vice versa.

## Future directions

Sophisticated labeling techniques as well as super-resolution microscopy enabled direct visualization of nuclear processes at several length scales, mostly well below the diffraction limit. By incorporation of data derived from orthogonal approaches, a more and more complete picture of chromatin structure (blob forming), dynamics, and function emerges. However, it must be kept in mind that the nucleus is a technically challenging organelle to analyze. Its content is highly crowded, and there are only few well-structured elements in the nucleus, which would facilitate the description of its interior by serving as reference points. Chromatin is a disordered, extraordinarily long, and confined biopolymer, which is constantly remodeled by a multitude of factors acting on it and that renders the analysis of chromatin and chromatin-associated processes highly complex.

Nevertheless, using advanced fluorescence imaging led us to a general picture in which transcription occurs in “transcription factories,” condensates likely formed by liquid-liquid phase separation, to which chromatin is tethered and thus globally and locally constrained. Transcription appears in various aspects to be a key process to take into account in order to understand the dynamic chromatin landscape, even though other factors must also contribute [1].

However, the dependency between chromatin architecture and transcription regulation in vivo is a matter of debate. For instance, contacts between *cis-*regulatory elements precede gene activation during *Drosophila* development and remain regardless of the transcriptional state [169]. A few studies also found that enhancer-promoter proximity is not always indicative of the transcriptional state [94, 170]. Strikingly, auxin-inducible degradation of the insulator protein CTCF, which acts together with cohesin in human cells to shape chromatin loop domains/TADs, has only a mild impact on transcriptional deregulation [171], and auxin-induced degradation of cohesin has a similarly modest effect on transcription [172]. However, several lines of research demonstrated that the deletion of TAD boundaries can perturb transcription by interfering with ectopic enhancer-promoter contacts [173–175]. Genes which are deregulated upon loss of cohesin or CTCF are preferentially located at TAD boundaries, hinting toward a subtle regulatory role of factors shaping the genomic environment around *cis-*regulatory elements. Transient looping events by cohesin and/or TFs, which are likely not to be detectable by ensemble approaches such as Hi-C, could thus constitute a flexible mechanism by which genes can be both activated and repressed, for instance, as a response to stimuli.

To further elucidate how chromatin and transcription are regulated, four main challenges remain in the development of live-cell real-time imaging of nuclear processes and chromatin behavior: (i) sequence-specific labeling, (ii) multicolor imaging, (iii) super-resolution microscopy techniques to allow rapid imaging in three dimensions at a nanoscale resolution of densely distributed emitters, and (iv) improving computational tools that allow processing and visualization of a large pool of information in order to understand stochastic processes.

At the single locus level, labeling technologies for tagging of multiple non-repetitive sequences to target specific chromatin domains for live-cell imaging need to be further improved to link chromatin dynamics to function. Applying a lattice light-sheet [176] illumination would be a technical solution to enhance image acquisition speed and decreasing phototoxicity [177, 178]. In addition, machine learning algorithms offer a computational solution for processing and denoising of “low-quality” images acquired rapidly [12]. Multicolor live-cell imaging to directly visualize proteins and chromatin at the nanoscale and short time resolution will be a major feat.

## Supplementary Information

**Additional file 1.** Review history.

## References

[1] van Steensel B, Furlong EEM. The role of transcription in shaping the spatial organization of the genome. Nat Rev Mol Cell Biol. 2019;20(6):327-37. 10.1038/s41580-019-0114-6 PMC7116054 30886333

[2] Gibcus JH, Dekker J. The hierarchy of the 3D genome. Mol Cell. 2013;49(5):773-82. 10.1016/j.molcel.2013.02.011 PMC3741673 23473598

[3] Lanctôt C, Cheutin T, Cremer M, Cavalli G, Cremer T. Dynamic genome architecture in the nuclear space: regulation of gene expression in three dimensions. Nat Rev Genet. 2007;8(2):104-15. 10.1038/nrg2041 17230197

[4] Bancaud A, Huet S, Daigle N, Mozziconacci J, Beaudouin J, Ellenberg J (2009). Molecular crowding affects diffusion and binding of nuclear proteins in heterochromatin and reveals the fractal organization of chromatin. EMBO J.

[5] Liu L, Shi G, Thirumalai D, Hyeon C. Chain organization of human interphase chromosome determines the spatiotemporal dynamics of chromatin loci. PLoS Comput Biol. 2018;14(12):e1006617. 10.1371/journal.pcbi.1006617 PMC6292649 30507936

[6] Dekker J, Rippe K, Dekker M, Kleckner N. Capturing chromosome conformation. Science. 2002;295(5558):1306-11. 10.1126/science.1067799 11847345

[7] Lieberman-Aiden E, van Berkum NL, Williams L, Imakaev M, Ragoczy T, Telling A, et al. Comprehensive mapping of long-range interactions reveals folding principles of the human genome. Science. 2009;326(5950):289-93. 10.1126/science.1181369 PMC2858594 19815776

[8] Shaban HA, Seeber A. Monitoring the spatio-temporal organization and dynamics of the genome. Nucleic Acids Res. 2020;48(7):3423-34. 10.1093/nar/gkaa135 PMC7144944 32123910

[9] Agbleke AA, Amitai A, Buenrostro JD, Chakrabarti A, Chu L, Hansen AS, et al. Advances in chromatin and chromosome research: perspectives from multiple fields. Mol Cell. 2020;79(6):881-901. 10.1016/j.molcel.2020.07.003 PMC7888594 32768408

[10] Boettiger A, Murphy S. Advances in chromatin imaging at kilobase-scale resolution. Trends Genet. 2020;36(4):273-87. 10.1016/j.tig.2019.12.010 PMC7197267 32007290

[11] Lakadamyali M, Cosma MP. Visualizing the genome in high resolution challenges our textbook understanding. Nat Methods. 2020;17(4):371-9. 10.1038/s41592-020-0758-3 32123395

[12] Barth R, Bystricky K, Shaban HA. Coupling chromatin structure and dynamics by live super-resolution imaging. Sci Adv. American Association for the Advancement of Science; 2020;6(27):eaaz2196. 10.1126/sciadv.aaz2196 PMC7458449 32937447

[13] Barth R, Fourel G, Shaban HA (2020). Dynamics as a cause for the nanoscale organization of the genome. Nucleus..

[14] Saintillan D, Shelley MJ, Zidovska A. Extensile motor activity drives coherent motions in a model of interphase chromatin. Proc Natl Acad Sci U S A. 2018;115(45):11442-7. 10.1073/pnas.1807073115 PMC6233076 30348795

[15] Shaban HA, Barth R, Bystricky K. Formation of correlated chromatin domains at nanoscale dynamic resolution during transcription. Nucleic Acids Res. 2018;46(13):e77. 10.1093/nar/gky269. 10.1093/nar/gky269 PMC6061878 29718294

[16] Nagashima R, Hibino K, Ashwin SS, Babokhov M, Fujishiro S, Imai R, et al. Single nucleosome imaging reveals loose genome chromatin networks via active RNA polymerase II. J Cell Biol. 2019;218(5):1511-30. 10.1083/jcb.201811090 PMC6504897 30824489

[17] Di Pierro M, Potoyan DA, Wolynes PG, Onuchic JN. Anomalous diffusion, spatial coherence, and viscoelasticity from the energy landscape of human chromosomes. Proc Natl Acad Sci. 2018;115(30):7753-8. 10.1073/pnas.1806297115 PMC6065008 29987017

[18] Cook PR, Marenduzzo D. Transcription-driven genome organization: a model for chromosome structure and the regulation of gene expression tested through simulations. Nucleic Acids Res. 2018;46(19):9895-906. 10.1093/nar/gky763 PMC6212781 30239812

[19] Tortora MM, Salari H, Jost D. Chromosome dynamics during interphase: a biophysical perspective. Curr Opin Genet Dev. 2020;61:37-43. 10.1016/j.gde.2020.03.001 32304901

[20] Dixon JR, Selvaraj S, Yue F, Kim A, Li Y, Shen Y (2012). Topological domains in mammalian genomes identified by analysis of chromatin interactions. Nature..

[21] Nora EP, Lajoie BR, Schulz EG, Giorgetti L, Okamoto I, Servant N (2012). Spatial partitioning of the regulatory landscape of the X-inactivation centre. Nature..

[22] Fortin JP, Hansen KD. Reconstructing A/B compartments as revealed by Hi-C using long-range correlations in epigenetic data. Genome Biol. 2015;16(1):180. 10.1186/s13059-015-0741-y PMC4574526 26316348

[23] Nagano T, Lubling Y, Stevens TJ, Schoenfelder S, Yaffe E, Dean W (2013). Single-cell Hi-C reveals cell-to-cell variability in chromosome structure. Nature..

[24] Nagano T, Lubling Y, Várnai C, Dudley C, Leung W, Baran Y (2017). Cell-cycle dynamics of chromosomal organization at single-cell resolution. Nature..

[25] Flyamer IM, Gassler J, Imakaev M, Brandão HB, Ulianov SV, Abdennur N, et al. Single-nucleus Hi-C reveals unique chromatin reorganization at oocyte-to-zygote transition. Nature. 2017;544(7648):110-4. 10.1038/nature21711 PMC5639698 28355183

[26] Stevens TJ, Lando D, Basu S, Liam P, Cao Y, Lee SF (2017). 3D structures of individual mammalian genomes studied by single-cell Hi-C. Nature Nature Publishing Group.

[27] Cattoni DI, Gizzi AMC, Georgieva M, Di Stefano M, Valeri A, Chamousset D, et al. Single-cell absolute contact probability detection reveals chromosomes are organized by multiple low-frequency yet specific interactions. Nat Commun. 2017;8(1):1-0. 10.1038/s41467-017-01962-x PMC5700980 29170434

[28] Fudenberg G, Imakaev M (2017). FISH-ing for captured contacts: towards reconciling FISH and 3C. Nat Methods.

[29] Fraser J, Williamson I, Bickmore WA, Dostie J. An overview of genome organization and how we got there: from FISH to Hi-C. Microbiol Mol Biol Rev. 2015;79(3):347-72. 10.1128/MMBR.00006-15 PMC4517094 26223848

[30] Giorgetti L, Heard E. Closing the loop: 3C versus DNA FISH. Genome Biol. 2016;17(1):1-9. 10.1186/s13059-016-1081-2 PMC5072311 27760553

[31] Wang S, Su JH, Beliveau BJ, Bintu B, Moffitt JR, Wu CT, et al. Spatial organization of chromatin domains and compartments in single chromosomes. Science (80-). 2016;353:598–602. 10.1126/science.aaf8084 PMC4991974 27445307

[32] Williamson I, Berlivet S, Eskeland R, Boyle S, Illingworth RS, Paquette D, et al. Spatial genome organization: contrasting views from chromosome conformation capture and fluorescence in situ hybridization. Genes Dev. 2014;28(24):2778-91. 10.1101/gad.251694.114 PMC4265680 25512564

[33] Shachar S, Voss TC, Pegoraro G, Sciascia N, Misteli T. Identification of gene positioning factors using high-throughput imaging mapping. Cell. 2015;162(4):911-23. 10.1016/j.cell.2015.07.035 PMC4538709 26276637

[34] Finn EH, Pegoraro G, Brandão HB, Valton AL, Oomen ME, Dekker J, et al. Extensive heterogeneity and intrinsic variation in spatial genome organization. Cell. 2019;176:1502–1515.e10. 10.1016/j.cell.2019.01.020 PMC6408223 30799036

[35] Su J-H, Zheng P, Kinrot SS, Bintu B, Zhuang X. Genome-scale imaging of the 3D organization and transcriptional activity of chromatin. Cell. 2020;182(6):1641-59. 10.1016/j.cell.2020.07.032 PMC7851072 32822575

[36] Kumari K, Duenweg B, Padinhateeri R, Prakash JR. Computing 3D chromatin configurations from contact probability maps by inverse Brownian dynamics. Biophys J. 2020;118(9):2193-2208. 10.1016/j.bpj.2020.02.017 PMC7203009 32389215

[37] Fudenberg G, Mirny LA (2012). Higher-order chromatin structure: bridging physics and biology. Curr Opin Genet Dev Elsevier Ltd.

[38] Van de Corput MPC, De Boer E, Knoch TA, Van Cappellen WA, Quintanilla A, Ferrand L, et al. Super-resolution imaging reveals three-dimensional folding dynamics of the β-globin locus upon gene activation. J Cell Sci. 2012;125(19):4630-9. 10.1242/jcs.108522 22767512

[39] Maass PG, Barutcu AR, Shechner DM, Weiner CL, Melé M, Rinn JL. Spatiotemporal allele organization by allele-specific CRISPR live-cell imaging (SNP-CLING). Nat Struct Mol Biol. 2018;25(2):176-84. 10.1038/s41594-017-0015-3 PMC5805655 29343869

[40] Wang H, Xu X, Nguyen CM, Liu Y, Gao Y, Lin X, et al. CRISPR-mediated programmable 3D genome positioning and nuclear organization. Cell. 2018;175(5):1405-17. 10.1016/j.cell.2018.09.013 PMC6239909 30318144

[41] Bintu B, Mateo LJ, Su JH, Sinnott-Armstrong NA, Parker M, Kinrot S, et al. Super-resolution chromatin tracing reveals domains and cooperative interactions in single cells. Science. 2018;362(6413). 10.1126/science.aau1783 PMC6535145 30361340

[42] Cardozo Gizzi AM, Cattoni DI, Fiche JB, Espinola SM, Gurgo J, Messina O, et al. Microscopy-based chromosome conformation capture enables simultaneous visualization of genome organization and transcription in intact organisms. Mol Cell. 2019;74:212–222.e5. 10.1016/j.molcel.2019.01.011 30795893

[43] Mateo LJ, Murphy SE, Hafner A, Cinquini IS, Walker CA, Boettiger AN. Visualizing DNA folding and RNA in embryos at single-cell resolution. Nature. 2019;568(7750):49-54. 10.1038/s41586-019-1035-4 PMC6556380 30886393

[44] Larson DR. Structure and function in Drosophila chromosomes: visualizing topological domains. Mol Cell. 2019;74(1):3-4. 10.1016/j.molcel.2019.03.017 30951650

[45] Lubeck E, Cai L. Single-cell systems biology by super-resolution imaging and combinatorial labeling. Nat Methods. 2012;9(7):743-8. 10.1038/nmeth.2069 PMC3418883 22660740

[46] Chen KH, Boettiger AN, Moffitt JR, Wang S, Zhuang X. Spatially resolved, highly multiplexed RNA profiling in single cells. Science. 2015;348(6233). 10.1126/science.aaa6090 PMC4662681 25858977

[47] Krietenstein N, Abraham S, Venev SV, Abdennur N, Gibcus J, Hsieh THS, et al. Ultrastructural details of mammalian chromosome architecture. Mol Cell. 2020;78(3):554-565.e7. 10.1016/j.molcel.2020.03.003 PMC7222625 32213324

[48] Hsieh THS, Weiner A, Lajoie B, Dekker J, Friedman N, Rando OJ. Mapping nucleosome resolution chromosome folding in yeast by micro-C. Cell. 2015;162(1):108-19. 10.1016/j.cell.2015.05.048 PMC4509605 26119342

[49] Ricci MA, Manzo C, García-Parajo MF, Lakadamyali M, Cosma MP (2015). Chromatin fibers are formed by heterogeneous groups of nucleosomes in vivo. Cell..

[50] Xu J, Ma H, Jin J, Uttam S, Fu R, Huang Y, et al. Super-resolution imaging of higher-order chromatin structures at different epigenomic states in single mammalian cells. Cell Rep. 2018;24(4):873-82. 10.1016/j.celrep.2018.06.085 PMC6154382 30044984

[51] Miron E, Oldenkamp R, Pinto DMS, Brown JM, Faria AR, Shaban HA, et al. Chromatin arranges in filaments of blobs with nanoscale functional zonation.Science Advances. 2020; 6(39):eaba8811. 10.1126/sciadv.aba8811 PMC7531892 32967822

[52] Bancaud A, Gadal O, Bystricky K. Capturing chromosome structural properties from their spatial and temporal fluctuations. Epigenetics and Systems Biology. Academic Press, 2017. 239-263.

[53] Seeber A, Hauer MH, Gasser SM. Chromosome dynamics in response to DNA damage. Annu Rev Genet. 2018;52:295–319. 10.1146/annurev-genet-120417-031334 30208290

[54] Robinett CC, Straight A, Li G, Willhelm C, Sudlow G, Murray A, et al. In vivo localization of DNA sequences and visualization of large-scale chromatin organization using lac operator/repressor recognition. J Cell Biol. 1996;135(6):1685-700. 10.1083/jcb.135.6.1685 PMC2133976 8991083

[55] Marshall WF, Straight A, Marko JF, Swedlow J, Dernburg A, Belmont A, et al. Interphase chromosomes undergo constrained diffusional motion in living cells. Curr Biol. 1997;7(12):930-9. 10.1016/s0960-9822(06)00412-x 9382846

[56] Heun P. Chromosome dynamics in the yeast interphase nucleus. Science. 2001;294(5549):2181-6. 10.1126/science.1065366 11739961

[57] Cabal GG, Genovesio A, Rodriguez-Navarro S, Zimmer C, Gadal O, Lesne A (2006). SAGA interacting factors confine sub-diffusion of transcribed genes to the nuclear envelope. Nature..

[58] Saad H, Gallardo F, Dalvai M, Tanguy-le-Gac N, Lane D, Bystricky K. DNA dynamics during early double-strand break processing revealed by non-intrusive imaging of living cells. PLoS Genet. 2014;10(3):e1004187. 10.1371/journal.pgen.1004187 PMC3952824 24625580

[59] Germier T, Kocanova S, Walther N, Elien Bancaud A, Shaban HAHA, Sellou H (2017). Real-time imaging of a single gene reveals transcription-initiated local confinement. Biophys J.

[60] Miyanari Y, Ziegler-Birling C, Torres-Padilla M-E. Live visualization of chromatin dynamics with fluorescent TALEs. Nat Struct Mol Biol. 2013;20(11):1321–4. . 10.1038/nsmb.2680 24096363

[61] Ma H, Reyes-Gutierrez P, Pederson T. Visualization of repetitive DNA sequences in human chromosomes with transcription activator-like effectors. Proc Natl Acad Sci. 2013;110(52):21048–53. 10.1073/pnas.1319097110 PMC3876203 24324157

[62] Chen B, Gilbert LA, Cimini BA, Schnitzbauer J, Zhang W, Li GW (2013). Dynamic imaging of genomic loci in living human cells by an optimized CRISPR/Cas system. Cell..

[63] Ma H, Naseri A, Reyes-Gutierrez P, Wolfe SA, Zhang S, Pederson T. Multicolor CRISPR labeling of chromosomal loci in human cells. Proc Natl Acad Sci U S A. 2015;112(10):3002-7. 10.1073/pnas.1420024112 PMC4364232 25713381

[64] Gu B, Swigut T, Spencley A, Bauer MR, Chung M, Meyer T, et al. Transcription-coupled changes in nuclear mobility of mammalian cis-regulatory elements. Science. 2018;359(6379):1050-5. 10.1126/science.aao3136 PMC6590518 29371426

[65] Bronshtein I, Kepten E, Kanter I, Berezin S, Lindner M, Redwood AB, et al. Loss of lamin A function increases chromatin dynamics in the nuclear interior. Nat Commun. 2015;6(1):1-9. 10.1038/ncomms9044 PMC4560783 26299252

[66] Chubb JR, Boyle S, Perry P, Bickmore WA (2002). Chromatin motion is constrained by association with nuclear compartments in human cells. Curr Biol.

[67] Zidovska A, Weitz DA, Mitchison TJ. Micron-scale coherence in interphase chromatin dynamics. Proc Natl Acad Sci U S A. 2013;110:15555–60. 10.1073/pnas.1220313110 PMC3785772 24019504

[68] Shinkai S, Nozaki T, Maeshima K, Togashi Y. Dynamic nucleosome movement provides structural information of topological chromatin domains in living human cells. PLoS Comput Biol. 2016;12(10):e1005136. 10.1371/journal.pcbi.1005136 PMC5072619 27764097

[69] Chuang CH, Carpenter AE, Fuchsova B, Johnson T, de Lanerolle P, Belmont AS (2006). Long-range directional movement of an interphase chromosome site. Curr Biol.

[70] Levi V, Ruan Q, Plutz M, Belmont AS, Gratton E. Chromatin dynamics in interphase cells revealed by tracking in a two-photon excitation microscope. Biophys J. 2005;89:4275–85. 10.1529/biophysj.105.066670 PMC1366992 16150965

[71] Chuang CH, Belmont AS. Moving chromatin within the interphase nucleus-controlled transitions? In Seminars in cell & developmental biology 2007 (Vol. 18, No. 5, pp. 698-706). Academic Press. 10.1016/j.semcdb.2007.08.012 PMC2117624 17905613

[72] Shaban HA, Barth R, Recoules L, Bystricky K. Hi-D: nanoscale mapping of nuclear dynamics in single living cells. Genome Biol. 2020;21:1-21. 10.1186/s13059-020-02002-6 PMC7168861 32312289

[73] Ellenberg J, Lippincott-Schwartz J, Presley JF. Dual-colour imaging with GFP variants. Trends Cell Biol. 1999;9(2):52-6. 10.1016/s0962-8924(98)01420-2 10087618

[74] Salic A, Mitchison TJ. A chemical method for fast and sensitive detection of DNA synthesis in vivo. Proc Natl Acad Sci U S A. 2008;105(7):2415-20. 10.1073/pnas.0712168105 PMC2268151 18272492

[75] Nozaki T, Imai R, Tanbo M, Nagashima R, Tamura S, Tani T. Dynamic organization of chromatin domains revealed by super-resolution live-cell imaging. Mol Cell. 2017;10:1–12. 10.1016/j.molcel.2017.06.018 28712725

[76] Lukinavičius G, Blaukopf C, Pershagen E, Schena A, Derivery E, Gonzalez-gaitan M, et al. A far-red DNA stain for live-cell nanoscopy. Nat Commun. 2015;61:3–5. 10.1038/ncomms9497 PMC4600740 26423723

[77] Bucevičius J, Keller-Findeisen J, Gilat T, Hell SW, Lukinavičius G. Rhodamine-Hoechst positional isomers for highly efficient staining of heterochromatin. Chem Sci. 2019;10(7):1962-70. 10.1039/c8sc05082a PMC6385482 30881625

[78] Scipioni L, Di Bona M, Vicidomini G, Diaspro A, Lanzanò L. Local raster image correlation spectroscopy generates high-resolution intracellular diffusion maps. Commun Biol. 2018;1:10. 10.1038/s42003-017-0010-6 PMC6053083 30271897

[79] Hinde E, Cardarelli F, Digman MA, Gratton E. In vivo pair correlation analysis of EGFP intranuclear diffusion reveals DNA-dependent molecular flow. Proc Natl Acad Sci. 2010;107:16560–5. 10.1073/pnas.1006731107 PMC2944750 20823232

[80] Liu Z, Tjian R. Visualizing transcription factor dynamics in living cells. J. Cell Biol. 2018;217(4):1181-91. 10.1083/jcb.201710038 PMC5881510 29378780

[81] McNally JC, Müller WG, Walker D, Wolford R, Hager GL. The glucocorticoid receptor: rapid exchange with regulatory sites in living cells. Science. 2000;287:1262–5. 10.1126/science.287.5456.1262 10678832

[82] Zhao ZW, White MD, Bissiere S, Levi V, Plachta N. Quantitative imaging of mammalian transcriptional dynamics: from single cells to whole embryos. BMC Biol. 2016;14(1):1-11. 10.1186/s12915-016-0331-9 PMC5180410 28010727

[83] Stenoien DL, Patel K, Mancini MG, Dutertre M, Smith CL, O’Malley BW (2001). FRAP reveals that mobility of oestrogen receptor-α is ligand- and proteasome-dependent. Nat Cell Biol.

[84] Gebhardt JCM, Suter DM, Roy R, Zhao ZW, Chapman AR, Basu S, et al. Single-molecule imaging of transcription factor binding to DNA in live mammalian cells. Nat Methods. 2013;10:421–6. 10.1038/nmeth.2411 PMC3664538 23524394

[85] Shechtman Y, Gustavsson A-K, Petrov PN, Dultz E, Lee MY, Weis K, et al. Observation of live chromatin dynamics in cells via 3D localization microscopy using tetrapod point spread functions. Biomed Opt Express. 2017;8:5735. 10.1364/BOE.8.005735 PMC5745116 29296501

[86] Michelman-Ribeiro A, Mazza D, Rosales T, Stasevich TJ, Boukari H, Rishi V (2009). Direct measurement of association and dissociation rates of DNA binding in live cells by fluorescence correlation spectroscopy. Biophys J.

[87] Chen J, Zhang Z, Li L, Chen BC, Revyakin A, Hajj B (2014). Single-molecule dynamics of enhanceosome assembly in embryonic stem cells. Cell..

[88] Suter DM. Transcription factors and DNA play hide and seek. Trends Cell Biol. 2020;30(6):491-500. 10.1016/j.tcb.2020.03.003 32413318

[89] Vukojević V, Papadopoulos DK, Terenius L, Gehring WJ, Rigler R. Quantitative study of synthetic Hox transcription factor-DNA interactions in live cells. Proc Natl Acad Sci U S A. 2010;107(9):4093-8. 10.1073/pnas.0914612107 PMC2840106 20147625

[90] Raccaud M, Friman ET, Alber AB, Agarwal H, Deluz C, Kuhn T, et al. Mitotic chromosome binding predicts transcription factor properties in interphase. Nat Commun. 2019;10(1):1-6. 10.1038/s41467-019-08417-5 PMC6353955 30700703

[91] Clauß K, Popp AP, Schulze L, Hettich J, Reisser M, Torres LE, et al. DNA residence time is a regulatory factor of transcription repression. Nucleic Acids Res. 2017;45(19):11121-30. 10.1093/nar/gkx728 PMC5737411 28977492

[92] Friman ET, Deluz C, Meireles-Filho ACA, Govindan S, Gardeux V, Deplancke B, et al. Dynamic regulation of chromatin accessibility by pluripotency transcription factors across the cell cycle. Elife. 2019;8:e50087. 10.7554/eLife.50087 PMC6890464 31794382

[93] Liu H, Dong P, Ioannou MS, Li L, Shea J, Pasolli HA, et al. Visualizing long-term single-molecule dynamics in vivo by stochastic protein labeling. Proc Natl Acad Sci. 2018;115:343–8. 10.1073/pnas.1713895115 PMC5777047 29284749

[94] Alexander JM, Guan J, Li B, Maliskova L, Song M, Shen Y, et al. Live-cell imaging reveals enhancer-dependent sox2 transcription in the absence of enhancer proximity. Elife. 2019;8:e41769. 10.7554/eLife.41769 PMC6534382 31124784

[95] Ferreira J, Paolella G, Ramos C, Lamond AI (1997). Spatial organization of large-scale chromatin domains in the nucleus: a magnified view of single chromosome territories. J Cell Biol.

[96] Heinz S, Texari L, Hayes MGB, Urbanowski M, Chang MW, Givarkes N, et al. Transcription elongation can affect genome 3D structure. Cell. 2018;174(6):1522-36. 10.1016/j.cell.2018.07.047 PMC6130916 30146161

[97] Dekker J, Mirny L (2016). The 3D genome as moderator of chromosomal communication. Cell..

[98] Fraser P. Transcriptional control thrown for a loop. Curr Opin Genet Dev. 2006;16(5):490-5. 10.1016/j.gde.2006.08.002 16904310

[99] Hübner MR, Spector DL (2010). Chromatin dynamics. Annu Rev Biophys.

[100] Jackson DA, Hassan AB, Errington RJ, Cook PR (1993). Visualization of focal sites of transcription within human nuclei. EMBO J.

[101] Wansink DG, Schul W, Van der Kraan I, Van Steensel B, Van Driel R, De Jong L (1993). Fluorescent labeling of nascent RNA reveals transcription by RNA polymerase II in domains scattered throughout the nucleus. J Cell Biol.

[102] Ghamari A, van de Corput MPC, Thongjuea S, van Cappellen WA, van Ijcken W, van Haren J, et al. In vivo live imaging of RNA polymerase II transcription factories in primary cells. Genes Dev, 2013;27:767–777. 10.1101/gad.216200.113 PMC3639417 23592796

[103] Cook PR. The organization of replication and transcription. Science. 1999;284(5421):1790-5. 10.1126/science.284.5421.1790 10364545

[104] Papantonis A, Cook PR. Transcription factories: genome organization and gene regulation. Chem Rev. 2013;113(11):8683-705. 10.1021/cr300513p 23597155

[105] Palstra RJ, Simonis M, Klous P, Brasset E, Eijkelkamp B, de Laat W. Maintenane of long-ranage DNA interactions after inhibition of ongoing RNA polymerase II transcription. PLoS One. 2008;3(2):e1661. 10.1371/journal.pone.0001661 PMC2243019 18286208

[106] Mitchell JA, Fraser P. Transcription factories are nuclear subcompartments that remain in the absence of transcription. Genes Dev. 2008;22(1):20-5. 10.1101/gad.454008 PMC2151011 18172162

[107] Chen H, Levo M, Barinov L, Fujioka M, Jaynes JB, Gregor T. Dynamic interplay between enhancer–promoter topology and gene activity. Nat Genet. 2018;50(9):1296-303. 10.1038/s41588-018-0175-z PMC6119122 30038397

[108] Tsai A, Alves MR, Crocker J. Multi-enhancer transcriptional hubs confer phenotypic robustness. Elife. 2019;8:e45325. 10.7554/eLife.45325 PMC6650246 31294690

[109] Jonkers I, Lis JT. Getting up to speed with transcription elongation by RNA polymerase II. Nat Rev Mol Cell Biol. 2015;16(3):167-77. 10.1038/nrm3953 PMC4782187 25693130

[110] Cisse II, Izeddin I, Causse SZ, Boudarene L, Senecal A, Muresan L, et al. Real-time dynamics of RNA polymerase II clustering in live human cells. Science. 2013;341:664–7. 10.1126/science.1239053 23828889

[111] Chen X, Wei M, Zheng MM, Zhao J, Hao H, Chang L (2016). Study of RNA polymerase II clustering inside live-cell nuclei using Bayesian nanoscopy. ACS Nano.

[112] Cho WK, Spille JH, Hecht M, Lee C, Li C, Grube V, et al. Mediator and RNA polymerase II clusters associate in transcription-dependent condensates. Science (80-). 2018;361:412–5. 10.1126/science.aar4199 PMC6543815 29930094

[113] Alberti S, Gladfelter A, Mittag T. Considerations and challenges in studying liquid-liquid phase separation and biomolecular condensates. Cell. 2019;176(3):419-34. 10.1016/j.cell.2018.12.035 PMC6445271 30682370

[114] Banani SF, Lee HO, Hyman AA, Rosen MK. Biomolecular condensates: organizers of cellular biochemistry. Nat Rev Mol Cell Biol. 2017;18(5):285-98. 10.1038/nrm.2017.7 PMC7434221 28225081

[115] Boija A, Klein IA, Sabari BR, Dall’Agnese A, Coffey EL, Zamudio AV, et al. Transcription factors activate genes through the phase-separation capacity of their activation domains. Cell. 2018;175(7):1842-55. 10.1016/j.cell.2018.10.042 PMC6295254 30449618

[116] Sabari BR, Dall’Agnese A, Boija A, Klein IA, Coffey EL, Shrinivas K, et al. Coactivator condensation at super-enhancers links phase separation and gene control. Science. 2018;361(6400). 10.1126/science.aar3958 PMC6092193 29930091

[117] Boehning M, Dugast-Darzacq C, Rankovic M, Hansen AS, Yu T, Marie-Nelly H, et al. RNA polymerase II clustering through carboxy-terminal domain phase separation. Nat Struct Mol Biol. 2018;25(9):833-40. 10.1038/s41594-018-0112-y 30127355

[118] Kwon I, Kato M, Xiang S, Wu L, Theodoropoulos P, Mirzaei H (2013). Phosphorylation-regulated binding of RNA polymerase II to fibrous polymers of low-complexity domains. Cell..

[119] Chong S, Dugast-Darzacq C, Liu Z, Dong P, Dailey GM, Cattoglio C, et al. Imaging dynamic and selective low-complexity domain interactions that control gene transcription. Science. 2018;361(6400). 10.1126/science.aar2555 PMC6961784 29930090

[120] Allen BL, Taatjes DJ. The mediator complex: a central integrator of transcription. Nat Rev Mol Cell Biol. 2015;16(3):155-66. 10.1038/nrm3951 PMC4963239 25693131

[121] Cho WK, Jayanth N, English BP, Inoue T, Andrews JO, Conway W, et al. RNA polymerase II cluster dynamics predict mRNA output in living cells. Elife. 2016;5:e13617. 10.7554/eLife.13617 PMC4929003 27138339

[122] Wang X. Cairns MJ. Nucleic Acids Res: Yan J. Super-enhancers in transcriptional regulation and genome organization; 2019;47(22):11481-96. 10.1093/nar/gkz1038 PMC7145697 31724731

[123] Shin Y, Chang YC, Lee DSW, Berry J, Sanders DW, Ronceray P, et al. Liquid nuclear condensates mechanically sense and restructure the genome. Cell. 2018;175(6):1481-91. 10.1016/j.cell.2018.10.057 PMC6724728 30500535

[124] Novo CL, Javierre BM, Cairns J, Segonds-Pichon A, Wingett SW, Freire-Pritchett P, et al. Long-range enhancer interactions are prevalent in mouse embryonic stem cells and are reorganized upon pluripotent state transition. Cell Rep. 2018;22(10):2615-27. 10.1016/j.celrep.2018.02.040 PMC5863031 29514091

[125] Ryu J, Kim H, Yang D, Lee AJ, Jung I. A new class of constitutively active super-enhancers is associated with fast recovery of 3D chromatin loops. BMC Bioinformatics. 2019;20(3):25-36. 10.1186/s12859-019-2646-3 PMC6439976 30925856

[126] Cook PR, Marenduzzo D (2009). Entropic organization of interphase chromosomes. J Cell Biol.

[127] Cremer T, Cremer M, Hübner B, Strickfaden H, Smeets D, Popken J, et al. The 4D nucleome: evidence for a dynamic nuclear landscape based on co-aligned active and inactive nuclear compartments. FEBS Lett. 2015;9(20):2931-43. 10.1016/j.febslet.2015.05.037 26028501

[128] Walther N, Hossain MJ, Politi AZ, Koch B, Kueblbeck M, Odegard-fougner Ø, et al. A quantitative map of human condensins provides new insights into mitotic chromosome architecture. J Cell Biol. 2018;217(7):2309-28. 10.1083/jcb.201801048 PMC6028534 29632028

[129] Fourel G, Magdinier F, Gilson É́. Insulator dynamics and the setting of chromatin domains. BioEssays. 2004;26(5):523-32. 10.1002/bies.20028 15112232

[130] Strom AR, Emelyanov AV, Mir M, Fyodorov DV, Darzacq X, Karpen GH. Phase separation drives heterochromatin domain formation. Nature. 2017;547(7662):241-5. 10.1038/nature22989 PMC6022742 28636597

[131] Sokolova E, Spruijt E, Hansen MMK, Dubuc E, Groen J, Chokkalingam V, et al. Enhanced transcription rates in membrane-free protocells formed by coacervation of cell lysate. Proc Natl Acad Sci U S A. 2013;110(29):11692-7. 10.1073/pnas.1222321110 PMC3718175 23818642

[132] Ge X, Luo D, Xu J. Cell-free protein expression under macromolecular crowding conditions. PLoS One. 2011;6(12):e28707. 10.1371/journal.pone.0028707 PMC3234285 22174874

[133] Chung SY, Lerner E, Jin Y, Kim S, Alhadid Y, Grimaud LW, et al. The effect of macromolecular crowding on single-round transcription by *Escherichia coli* RNA polymerase. Nucleic Acids Res. 2019;47(3):1440-50. 10.1093/nar/gky1277 PMC6379708 30590739

[134] Matsuda H, Putzel GG, Backman V, Szleifer I. Macromolecular crowding as a regulator of gene transcription. Biophys J. 2014;106(8):1801-10. 10.1016/j.bpj.2014.02.019 PMC4008821 24739179

[135] Shim AR, Nap RJ, Huang K, Almassalha LM, Matusda H, Backman V, et al. Dynamic crowding regulates transcription. BiophysJ. 2019;118(9):2117-29. 10.1016/j.bpj.2019.11.007 PMC7202930 31818468

[136] Socol M, Wang R, Jost D, Carrivain P, Vaillant C, Le Cam E, et al. Rouse model with transient intramolecular contacts on a timescale of seconds recapitulates folding and fluctuation of yeast chromosomes. Nucleic Acids Res. 2019;47(12):6195-207. 10.1093/nar/gkz374 PMC6614813 31114898

[137] Amitai A. Chromatin configuration affects the dynamics and distribution of a transiently interacting protein. Biophys J. 2018;114(4):766-71. 10.1016/j.bpj.2017.12.037 PMC5984998 29395046

[138] Weber SC, Spakowitz AJ, Theriot JA. Bacterial chromosomal loci move subdiffusively through a viscoelastic cytoplasm. Phys Rev Lett. 2010;104(23):238102. 10.1103/PhysRevLett.104.238102 PMC4929007 20867274

[139] Kim JS, Backman V, Szleifer I. Crowding-induced structural alterations of random-loop chromosome model. Phys Rev Lett. 2011;106(16):168102. 10.1103/PhysRevLett.106.168102 21599416

[140] Kang H, Pincus PA, Hyeon C, Thirumalai D. Effects of macromolecular crowding on the collapse of biopolymers. Phys Rev Lett. 2015;114(6):068303. 10.1103/PhysRevLett.114.068303 25723249

[141] Guérin T, Prost J, Martin P, Joanny JF. Coordination and collective properties of molecular motors: theory. Curr Opin Cell Biol. 2010;22(1):14-20. 10.1016/j.ceb.2009.12.012 20074926

[142] Holzbaur EL, Goldman YE. Coordination of molecular motors: from in vitro assays to intracellular dynamics. Curr Opin Cell Biol. 2010;22(1):4-13. 10.1016/j.ceb.2009.12.014 PMC2846361 20102789

[143] Put S, Sakaue T, Vanderzande C. Active dynamics and spatially coherent motion in chromosomes subject to enzymatic force dipoles. Phys Rev E. 2019;99(3):032421. 10.1103/PhysRevE.99.032421 30999440

[144] Di Pierro M, Zhang B, Aiden EL, Wolynes PG, Onuchic JN. Transferable model for chromosome architecture. Proc Natl Acad Sci. 2016;113(43):12168-73. 10.1073/pnas.1613607113 PMC5087044 27688758

[145] Sewitz SA, Fahmi Z, Aljebali L, Bancroft J, Brustolini OJB, Saad H, et al. Heterogeneous chromatin mobility derived from chromatin states is a determinant of genome organisation in *S. cerevisiae*. bioRxiv [Internet]. 2017;106344. Available from: https://www.biorxiv.org/content/early/2017/03/02/106344.full.pdf+html.

[146] Brackley CA, Taylor S, Papantonis A, Cook PR, Marenduzzo D. Nonspecific bridging-induced attraction drives clustering of DNA-binding proteins and genome organization. Proc Natl Acad Sci. 2013;110:E3605–11. 10.1073/pnas.1302950110 PMC3780866 24003126

[147] Liu Z, Legant WR, Chen BC, Li L, Grimm JB, Lavis LD (2014). 3D imaging of Sox2 enhancer clusters in embryonic stem cells. Elife..

[148] Brackley CA, Johnson J, Kelly S, Cook PR, Marenduzzo D (2016). Simulated binding of transcription factors to active and inactive regions folds human chromosomes into loops, rosettes and topological domains. Nucleic Acids Res.

[149] Barbieri M, Chotalia M, Fraser J, Lavitas LM, Dostie J, Pombo A, et al. Complexity of chromatin folding is captured by the strings and binders switch model. Proc Natl Acad Sci U S A. 2012;109(40):16173-8. 10.1073/pnas.1204799109 PMC3479593 22988072

[150] Alipour E, Marko JF (2012). Self-organization of domain structures by DNA-loop-extruding enzymes. Nucleic Acids Res.

[151] Riggs AD. DNA methylation and late replication probably aid cell memory, and type I DNA reeling could aid chromosome folding and enhancer function. Philos Trans R Soc Lond Ser B Biol Sci. 1990;326(1235):285-97. 10.1098/rstb.1990.0012 1968665

[152] Nasmyth K. Disseminating the genome: joining, resolving, and separating sister chromatids during mitosis and meiosis. Annu Rev Genet. 2001;35(1):673-745. 10.1146/annurev.genet.35.102401.091334 11700297

[153] Ganji M, Shaltiel IA, Bisht S, Kim E, Kalichava A, Haering CH, et al. Real-time imaging of DNA loop extrusion by condensin. Science. 2018;360(6384):102-5. 10.1126/science.aar7831 PMC6329450 29472443

[154] Stefan Golfier, Thomas Quail, Hiroshi Kimura JB. Cohesin and condensin extrude loops in a cell-cycle dependent manner. bioRxiv. 2019. 10.7554/eLife.53885 PMC7316503 32396063

[155] Kong M, Cutts EE, Pan D, Beuron F, Kaliyappan T, Xue C, et al. Human condensin I and II drive extensive ATP-dependent compaction of nucleosome-bound DNA. Mol Cell. 2020;79(1):99-114.e9. 10.1016/j.molcel.2020.04.026 PMC7335352 32445620

[156] Davidson IF, Bauer B, Goetz D, Tang W, Wutz G, Peters J-M. DNA loop extrusion by human cohesin. Science. 2019;366(6471):1338-45. 10.1126/science.aaz3418 31753851

[157] Kim Y, Shi Z, Zhang H, Finkelstein IJ, Yu H. Human cohesin compacts DNA by loop extrusion. Science. 2019;366(6471):1345-9. 10.1126/science.aaz4475 PMC7387118 31780627

[158] Fudenberg G, Imakaev M, Lu C, Goloborodko A, Abdennur N, Mirny LA. Formation of chromosomal domains by loop extrusion. Cell Rep. 2016;15(9):2038-49. 10.1016/j.celrep.2016.04.085 PMC4889513 27210764

[159] Sanborn AL, Rao SSP, Huang S-C, Durand NC, Huntley MH, Jewett AI, et al. Chromatin extrusion explains key features of loop and domain formation in wild-type and engineered genomes. Proc Natl Acad Sci. 2015;112(47):E6456-65. 10.1073/pnas.1518552112 PMC4664323 26499245

[160] Pereira MCF, Brackley CA, Michieletto D, Annunziatella C, Bianco S, Chiariello AM, et al. Complementary chromosome folding by transcription factors and cohesin. bioRxiv. 2018.

[161] Nuebler J, Fudenberg G, Imakaev M, Abdennur N, Mirny LA (2018). Chromatin organization by an interplay of loop extrusion and compartmental segregation. Proc Natl Acad Sci.

[162] Le TB, Laub MT. Transcription rate and transcript length drive formation of chromosomal interaction domain boundaries. EMBO J. 2016;35(14):1582-95. . 10.15252/embj.201593561 PMC4946140 27288403

[163] Beagrie RA, Scialdone A, Schueler M, Kraemer DCA, Chotalia M, Xie SQ (2017). Complex multi-enhancer contacts captured by genome architecture mapping. Nature..

[164] Olivares-Chauvet P, Mukamel Z, Lifshitz A, Schwartzman O, Elkayam NO, Lubling Y, et al. Capturing pairwise and multi-way chromosomal conformations using chromosomal walks. Nature. 2016;540(7632):296-300. 10.1038/nature20158 27919068

[165] Fullwood MJ, Liu MH, Pan YF, Liu J, Xu H, Mohamed Y Bin, et al. An oestrogen-receptor-α-bound human chromatin interactome. Nature. 2009;462(7269):58-64. . 10.1038/nature08497 PMC2774924 19890323

[166] Davidson IF, Bauer B, Goetz D, Tang W, Wutz G, Peters J-M. DNA loop extrusion by human cohesin. Science. 2019;366(6471):1338-45. . 10.1126/science.aaz3418 31753851

[167] Golfier S, Quail T, Kimura H, Brugués J. Cohesin and condensin extrude DNA loops in a cell-cycle dependent manner. Elife. 2020;9:e53885. . 10.7554/eLife.53885 PMC7316503 32396063

[168] Brandão HB, Paul P, van den Berg AA, Rudner DZ, Wang X, Mirny LA (2019). RNA polymerases as moving barriers to condensin loop extrusion. Proc Natl Acad Sci.

[169] Espinola SM, Götz M, Fiche J-B, Bellec M, Houbron C, Cardozo Gizzi AM, et al. *Cis*-regulatory chromatin loops arise before TADs and gene activation, and are independent of cell fate during development. bioRxiv [Internet]. 2020;2020.07.07.191015. Available from: http://biorxiv.org/content/early/2020/07/07/2020.07.07.191015.abstract. 10.1038/s41588-021-00816-z 33795867

[170] Schoenfelder S, Fraser P. Long-range enhancer–promoter contacts in gene expression control. Nat Rev Genet. 2019;1. 10.1038/s41576-019-0128-0 31086298

[171] Nora EP, Goloborodko A, Valton AL, Gibcus JH, Uebersohn A, Abdennur N, et al. Targeted degradation of CTCF decouples local insulation of chromosome domains from genomic compartmentalization. Cell. 2017;169(5):930-44. 10.1016/j.cell.2017.05.004 PMC5538188 28525758

[172] Rao SSP, Huang SC, Glenn St Hilaire B, Engreitz JM, Perez EM, Kieffer-Kwon KR, et al. Cohesin loss eliminates all loop domains. Cell. 2017;171(2):305-20. 10.1016/j.cell.2017.09.026 PMC5846482 28985562

[173] Guo Y, Xu Q, Canzio D, Shou J, Li J, Gorkin DU, et al. CRISPR inversion of CTCF sSites alters genome topology and enhancer/promoter function. Cell. 2015;162(4):900-10. 10.1016/j.cell.2015.07.038 PMC4642453 26276636

[174] Lupiáñez DG, Kraft K, Heinrich V, Krawitz P, Brancati F, Klopocki E, et al. Disruptions of topological chromatin domains cause pathogenic rewiring of gene-enhancer interactions. Cell. 2015;161(5):1012-25. 10.1016/j.cell.2015.04.004 PMC4791538 25959774

[175] Hnisz D, Weintrau AS, Day DS, Valton AL, Bak RO, Li CH, et al. Activation of proto-oncogenes by disruption of chromosome neighborhoods. Science. 2016;351(6280):1454-8. 10.1126/science.aad9024 PMC4884612 26940867

[176] Chen BC, Legant WR, Wang K, Shao L, Milkie DE, Davidson MW, et al. Lattice light-sheet microscopy: imaging molecules to embryos at high spatiotemporal resolution. Science. 2014;346(6208). 10.1126/science.1257998 PMC4336192 25342811

[177] Huang X, Fan J, Li L, Liu H, Wu R, Wu Y, et al. Fast, long-term, super-resolution imaging with Hessian structured illumination microscopy. Nat Biotechnol. 2018;36(5):451. 10.1038/nbt.4115 29644998

[178] Wu Y, Shroff H. Faster, sharper, and deeper: structured illumination microscopy for biological imaging. Nat Methods. 2018;15(12):1011-9. 10.1038/s41592-018-0211-z 30478322

